# Supplementary material for: Clinical outcomes of circumcisions and prevalence of complications of male circumcisions: A five-year retrospective analysis at a teaching hospital in Ghana
Source: PLOS Glob Public Health. 2025 Feb 3;5(2):e0003788. doi: 10.1371/journal.pgph.0003788 (PMC11790115; doi:10.1371/journal.pgph.0003788)
Supplement: Attachment — Submitted filename: revised circmcison response letter.pdf [file pgph.0003788.s002.pdf]

Dear Dr. Zimmerman,

Thank you for the opportunity to revise and resubmit our manuscript titled "Clinical Outcomes and Prevalence of Complications of Male Circumcisions: A Five-Year Retrospective Analysis at a Teaching Hospital in Ghana" (PGPH-D-24-02131). We appreciate the thoughtful feedback provided by both you and the reviewer. We have carefully addressed the points raised and made the necessary revisions. Below, I summarize our responses to the major comments.

1. Images:

As per the reviewer’s recommendation, we have removed the images from the manuscript as they were deemed unnecessary for answering the research question and supporting the results.

2. Author Summary:

We have added an Author Summary between the Abstract and Introduction sections. The summary is within the 150-200 word limit and aims to make our findings accessible to a broad audience, including both scientists and non-scientists.

4. General Comments and Title:

We have revised the manuscript to replace terms such as "mishap," "complications," and "catastrophe" with "adverse events," as recommended. Additionally, we have updated the title to better reflect the focus on circumcisions performed during the neonatal period and early infancy.

5. Introduction:

We have added information regarding the prevalence of circumcision-related adverse events in different contexts, including therapeutic circumcisions, voluntary medical male circumcisions for HIV prevention, and traditional circumcisions, to provide a broader context for the study.

6. Materials and Methods:

We have added a statement clarifying the potential limitation regarding the prevalence estimate of adverse events (AEs) due to the inclusion of only referrals from other facilities with AEs, and have noted this as a limitation in the manuscript.

7. Ethical Statement:

As the study was based on a retrospective review of data collected over the previous five years, we have clarified how consent was obtained for clients whose clinical records were included in the study.

8. Results:

- **Figure 1**: We have clarified that the reported numbers refer to Ho Teaching Hospital alone, not including the referring facilities within its catchment area.
- **Figure 2**: We have revised the title and narrative for clarity. The figure now presents both trends in circumcisions and salvage surgeries conducted at Ho Teaching Hospital, with improved clarity in the figure description.
- **Table 1**: We have revised Table 1 to clarify that the 44 patients listed may include multiple measurements for some individuals, and the table now aligns with the statement in the abstract regarding the number of complications.
- **Table 2**: We have added a summary of the framework used to classify circumcision-related adverse events as acute or cold, and we have edited the table caption for clarity. The key message regarding the timing of presentations for acute versus cold symptoms is now clearer.
- **Table 3**: We have clarified that the referral decision-makers and number of referrals may have included cases where individuals with multiple adverse events were referred by different decision-makers.
- **Table 4**: We have revised the table title and description to avoid confusion between coincidental diagnoses and circumcision-related complications. The focus is now clearly on adverse events related to circumcision, with coincidental diagnoses listed separately.
- **Tables 5 and 6**: We have merged Tables 5 and 6 as recommended and revised the caption to better reflect the key findings.

We trust that these revisions address the concerns raised and that the manuscript now meets the standards required for publication in PLOS Global Public Health. We look forward to your feedback on our revised manuscript.

Thank you once again for your consideration.

Sincerely,
Dr. Frank Obeng
